# Time-Resolved Fluorescence Immunoassay for C-Reactive Protein Using Colloidal Semiconducting Nanoparticles

**DOI:** 10.3390/s111211335

**Published:** 2011-11-28

**Authors:** Harri Härmä, Juha Toivonen, Juhani T. Soini, Pekka Hänninen, Wolfgang J. Parak

**Affiliations:** 1 Laboratory of Biophysics, University of Turku, 20520 Turku, Finland; E-Mails: juhani.soini@utu.fi (J.S.); pekka.hanninen@utu.fi (P.H.); 2 Department of Physics, Tampere University of Technology, 33101 Tampere, Finland; E-Mail: juha.toivonen@tut.fi (J.T.); 3 Turku University of Applied Sciences, 20520 Turku, Finland; 4 Fachbereich Physik and Wissenschaftliches Zentrum für Materialwissenschaften, Philipps Universität Marburg., 35037 Marburg, Germany; E-Mail: wolfgang.parak@physik.uni-marburg.de (W.P.)

**Keywords:** colloidal nanoparticles, quantum dots, time-gated fluorescence, sensing, bioaffinity assays

## Abstract

Besides the typical short-lived fluorescence with decay times in the nanosecond range, colloidal II/VI semiconductor nanoparticles dispersed in buffer also possess a long-lived fluorescence component with decay times in the microsecond range. Here, the signal intensity of the long-lived luminescence at microsecond range is shown to increase 1,000-fold for CdTe nanoparticles in PBS buffer. This long-lived fluorescence can be conveniently employed for time-gated fluorescence detection, which allows for improved signal-to-noise ratio and thus the use of low concentrations of nanoparticles. The detection principle is demonstrated with a time-resolved fluorescence immunoassay for the detection of C-reactive protein (CRP) using CdSe-ZnS nanoparticles and green light excitation.

## 1. Introduction

Time-resolved luminescence of lanthanide complexes has been applied extensively to diagnostic and drug discovery bioaffinity assays over the past decades [1]. The gated luminescence measurement significantly reduces autoluminescence and background luminescence in detection, leading to high assay sensitivity. Typically 3–4 orders of magnitude lower detection limits are measured using the lanthanide chemistry and time-resolved luminescence (a typical measurement: >50 μs delay time, >50 μs integration time, repetition rate 1 ms, 1 s total measurement time) compared to conventional fluorescence (a typical steady-state measurement: 1 s total measurement time using continuous excitation) [2]. Long-lived luminescence with time-gated measurements in the sub millisecond range has been reported also for nanomaterials [3,4]. Size-dependent fluorescence of semiconductor nanoparticles (NPs) has been extensively investigated over the years and good understanding of the structural and chemical properties of NP fluorescence has been obtained [5–7]. Typically, short-lived fluorescence signals with multiple decay rates below 500 ns range are detected for semiconductor NPs [8]. The rapidly decaying fluorescence of NPs can be readily affected by their surface properties. Surface capping of semiconductor NPs in aqueous medium has a major impact mainly for two reasons: colloidal stability and reduction of surface related defects. Loss of colloidal stability greatly reduces the rapidly decaying fluorescence signal of the semiconductor NPs, as they undergo self-quenching. Time-gated fluorescence measurement of semiconductor NPs has been also successfully demonstrated [9]. In this study we have manipulated and studied the long-lived fluorescence of semiconductor NPs and demonstrated the potential of long-lived NP fluorescence at the microsecond scale for bioaffinity assays using C-reactive protein as a model system.

## 2. Experimental Section

For the investigation of long-lived NP fluorescence differently sized mercaptopropionic acid capped CdTe NPs having fluorescence emission maxima at 592, 612, 676 and 728 (“NP728”) nm were prepared in aqueous phase [8] and used along with commercial streptavidin-coated CdSe-ZnS particles (Life Technologies, Carlsbad, CA, USA). Time-gated fluorescence detection was performed in a microtiter plate reader with 75 μs delay and 50 s integration times at 340 and 730 nm excitation and emission wavelengths, respectively.

A conventional sandwich-type immunoassay was developed on commercial streptavidin-labeled CdSe-ZnS core-shell NPs and time-resolved fluorescence detection. In order to benchmark the developed concept a parallel experiment using streptavidin conjugated with europium(III) chelate (Delfia Technique, PerkinElmer, Turku, Finland) as a luminescent label was applied. Anti-CRP monoclonal antibody mAb6405 (Medix Biochemica, Kauniainen, Finland) was immobilized on a microtiter well. After surface saturation with bovine serum albumin and washing, C-reactive protein was added to the wells for 30 min followed by a washing step. Biotinylated mAb6404 (Medix Biochemica) was incubated in the well for 30 min and europium(III)-labeled streptavidin or streptavidin-conjugated CdSe-ZnS were added after a washing step. During the final washing step, unbound labeled streptavidin was removed and the microtiter wells containing CdSe-ZnS NPs were measured for time-resolved fluorescence signals. Europium(III) enhancement solution was added to the wells containing europium-labeled streptavidin and the time-resolved luminescence signal was detected after 20 min incubation. The time-resolved fluorescence of semiconductor NPs was measured using an in-house built epi platefluorometer. A standard Fluoroscan Ascent microplate fluorometer (ThermoFisher, Helsinki, Finland) was modified by replacing the excitation light source with a 10 mW frequency-doubled diode-pumped solid-state Nd:YVO4 laser (532 nm, average cw power 10 mW) which was modulated to produce a pulse of 1 μs, integration time of 50 µs and 10 kHz repetition rate. Eu(III)-chelate labeled streptavidin was monitored using 400 μs delay and 400 μs integration times in a commercial microtiter plate reader as the instrument is optimized to measure long lifetime lanthanide luminescence.

## 3. Results and Discussion

**Figure 1.**
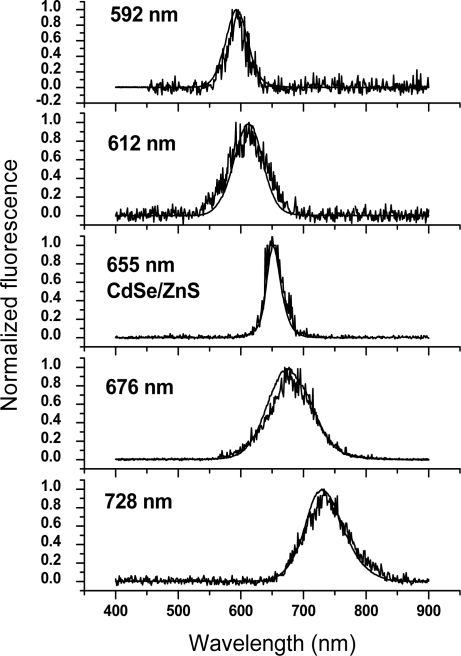
Short- (smooth, steady-state fluorescence) and long-lived (non-smooth, time-resolved luminescence) luminescence emission spectra of CdTe NPs of different size (emission maximum at 592, 615, 676 and 728 nm) and commercial CdSe/ZnS NPs (emission maximum at 655 nm) as recorded in PBS.

The detected conventional and time-gated fluorescence of the CdTe and CdSe/ZnS NP samples is shown in Figure 1.

**Figure 2.**
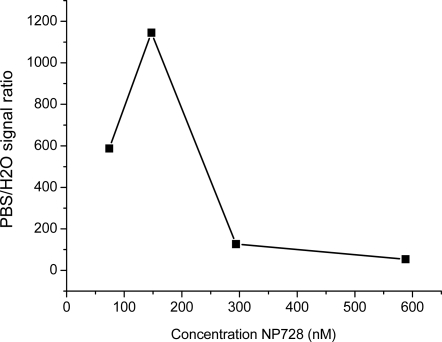
Long-lived fluorescence measured for NP728 in PBS and water at varying NP concentration. Incubation of NPs in PBS increases the long-lived fluorescence 1,000-fold compared to incubation in water. The signal-increase is NP-concentration dependent.

NP728 was the sample most studied by us. No significant long-lived fluorescence at the microsecond scale could be detected for NP728 suspended in water using time-gated fluorescence detection. However, by suspending the NPs in phosphate buffer saline (PBS) a 1,000-fold improvement in the gated long-lived fluorescence was observed (Figure 2). Long-lived luminescence of semiconductor NPs has been discussed before by several groups. Rossetti and Brus have studied CdS NPs coated with a ZnS semiconductor having a larger band-gap than CdS [10]. They speculated that ZnS may have the effect of preventing h + (hole) access to the water interface, and thus increase the h + lifetime. Holes are in solid state physics terminology positively charged vacancies in the valence band, which occur, for example, when electrons are excited from the valence to the conduction band. Spanhel *et al.* have discussed that, generally, the fluorescence close to the band gap energy decays fast and is an indication of band gap recombination [11].

**Figure 3.**
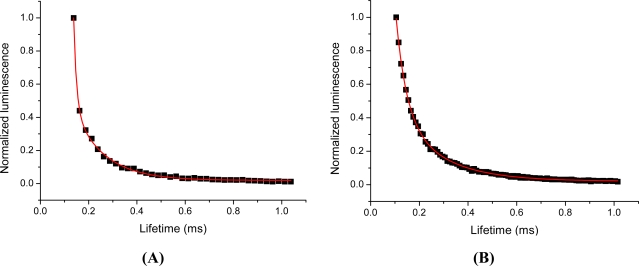
Fluorescence lifetimes were 178 and 42 μs for NP728 **(A)** and 126 and 12 μs for core-shell CdSe-ZnS **(B)**. The data was fitted to two-exponential decay function y = A_1_ × e(−k_1_ × t) + A_2_ × e(−k_2_ × t) where A is luminescence intensity, k is lifetime and t is time.

Long lifetime fluorescence at the band gap associated emission wavelength is believed to originate from charge carriers in weak traps, with less than 0.1 eV trap depth. A six-fold increase in the fluorescence lifetime has been also reported by overcoating of CdSe with CdS. Our observation for the 1,000-fold increase in the long-lived fluorescence signal may be attributed to a reduction in surface thiol capping leading to surface defects. Gradual loss of thiol capping due to photooxidation is a well known fact [12]. Another speculative mechanism might be associated with the onset of agglomeration of the CdTe NPs in salt-containing buffer [13]. This mechanism would however follow the loss of the thiol capping, as without appropriate capping NPs agglomerate [14]. The studied commercial streptavidin-coated CdSe-ZnS particles are apparently colloidally stable. This indicates that the mechanism may not be related to aggregation (which could have been the case in case of the CdTe NPs) but rather to surface properties of the semiconducting NPs. Independent of the mechanism, these measurements indicate that significant time-gated fluorescence can be detected at microsecond scale with semiconductor NPs. Using a standard time-gated spectrofluorometer, two-exponential lifetimes of 178 and 42 μs were measured for NP728 in PBS, see Figure 3.

Our initial observation on the long-lived fluorescence of CdTe led us to investigate long-lived luminescence of a more defined system and we switched to study commercial core-shell NPs, streptavidin-coated CdSe-ZnS having a 655 nm emission maximum (Life Technologies), on the microsecond scale. Lifetimes of 126 and 12 μs were measured for these commercial NPs, see Figure 3. The measured total fluorescence and time-resolved fluorescence of synthesized CdTe and CdSe-ZnS NPs were monitored rendering nearly perfectly overlapping emission spectra, see Figure 1. It is widely accepted that the short-lived emission luminescence is due to electron-hole pair radiative recombination from shallow trap states (near band gap recombination). Having the same excitation and emission spectra, the long-lived luminescence should originate from the very same shallow trap within the NPs. This suggests that additional energy transition levels can be excluded as an origin for the long-lived luminescence. As the spectral overlap of the excitation and emission wavelengths for the differently-sized semiconductor NPs were observed the spectral characteristics must be independent of the particle size and, thus, the emission wavelength.

**Figure 4.**
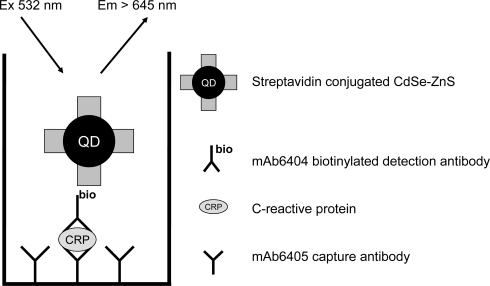
Principle of the C-reactive protein immunoassay. Antibody captures C-reactive protein to the microtiter well surface. After washing biotinylated detection antibody reacts with the surface-formed complex. Excess of detection antibody was removed and streptavidin-conjugated CdSe-ZnS particles detect the bound biotin correlating with the C-reactive protein concentration.

**Figure 5.**
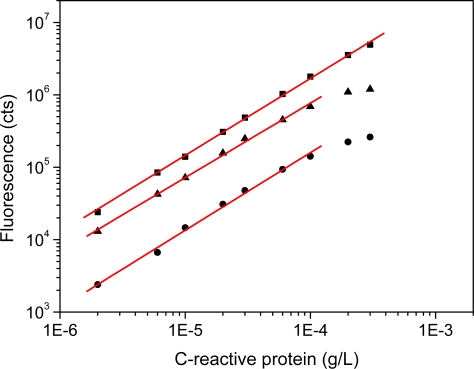
Calibration curves of two-site heterogeneous C-reactive protein immunoassay. C-reactive protein was detected using commercial CdSe/ZnS-labeled (short- (•) and long-lived (▴) fluorescence) and Eu(III)-labeled (▪) streptavidin.

Having been able to detect long-lived fluorescence for CdSe-ZnS NPs a conventional sandwich-type immunoassay based on time-resolved fluorescence detection was developed (Figure 4). We selected the CdSe-ZnS over prepared CdTe NPs because the commercial NPs carried a bioactive molecule for the immunoassay. Thus any issue regarding NP colloidal instability and thus uncontrolled signal was avoided. Performance of commercial streptavidin-labeled CdSe-ZnS core-shell NPs was compared to streptavidin conjugated with europium(III) chelate. C-reactive protein is the widely used rapid indicator for inflammation and thus chosen as a model system to demonstrate the functionality of the time-resolved luminescence detection with semiconductor nanoparticles. The calibration curves for the different detection modes are shown in Figure 5. The lowest limits of detection were 0.032, 0.55 and 0.47 μg/L for time-resolved luminescence of Eu(III), conventional, and time-resolved fluorescence of CdSe-ZnS, respectively. The coefficient of variation ranged from 1–11%, 2–6%, 2–6%, and curve parameters were:

$$\begin{array}{c} \text{y}\;=\;(1.06\pm 0.01)\;\text{x}\;+\;(10.5\pm 0.06),\;\text{R}\;=\;0.999;\\ \text{y}\;=\;(1.07\pm 0.05)\;\text{x}\;+\;(9.5\pm 0.24),\;\text{R}=0.996;\;\text{and}\\ \text{y }=\;(1.00\pm 0.03)\text{x}+(9.9\pm 0.15),\text{R}=0.998,\;\text{respectively}. \end{array}$$

A non-competitive immunoassay was successfully developed for C-reactive protein in a model assay system using semiconductor NPs as labels. A sensitivity of 0.47 μg/L was achieved in time-resolved fluorescence detection mode. The developed assay was still 10-fold less sensitive than that of the lanthanide(III) chelate–based method. Further studies must be conducted to reveal the full potential of time-resolved fluorescence detection using semiconductor NPs in respect to their surface properties and capping. Larger signal outcome and, therefore, improved specific activity of the label may be achieved by varying synthesis routes of semiconductor NPs. From the instrumental viewpoint, further improvement in the time-resolved detection mode can be achieved by careful optimization of optical components to reduce their long decay-time autofluorescence properties and a laser with shorter pulse length would further enhance the system sensitivity.

## References

[1] Hänninen P., Härmä H. (2011). Lanthanide Luminescence: Photophysical, Analytical and Biological Aspects. Springer Series on Fluorescence.

[2] Hemmila I., Mukkala V.M. (2001). Time-resolution in fluorometry technologies, labels, and applications in bioanalytical assays. Critical reviews. Clin. Lab. Sci.

[3] Beverloo H.B., van Schadewijk A., van Gelderen-Boele S., Tanke H.J. (1990). Inorganic phosphors as new luminescent labels for immunocytochemistry and time-resolved microscopy. Cytometry.

[4] Härmä H., Soukka T., Lövgren T. (2001). Europium nanoparticles and time-resolved fluorescence for ultrasensitive detection of prostate-specific antigen. Clin. Chem.

[5] Gaponik N., Hickey S.G., Dorfs D., Rogach A.L., Eychmüller A. (2010). Progress in the light emission of colloidal semiconductor nanocrystals. Small.

[6] Fu A., Gu W., Larabell C., Alivisatos A.P. (2005). Semiconductor nanocrystals for biological imaging. Curr. Opin. Neurobiol.

[7] Parak W.J., Pellegrino T., Plank C. (2005). Labelling of cells with quantum dots. Nanotechnology.

[8] Rogach A.L., Franzl T., Klar T.A., Feldmann J., Gaponik N., Lesnyak V., Shavel A., Eychmüller A., Rakovich Y.P., Donegan J.F. (2007). Aqueous synthesis of thiol-capped cdte nanocrystals: State-of-the-art. J. Phys. Chem. C.

[9] Dahan M., Laurence T., Pinaud F., Chemla D.S., Alivisatos A.P., Sauer M., Weiss S. (2001). Time-gated biological imaging by use of colloidal quantum dots. Opt. Lett.

[10] Rossetti R., Brus L. (1982). Electron-hole recombination emission as a probe of surface-chemistry in aqueous CdS colloids. J. Phys. Chem.

[11] Spanhel L., Haase M., Weller H., Henglein A. (1987). Photochemistry of colloidal semiconductors. 20. Surface modification and stability of strog luminescing CdS particles. J. Am. Chem. Soc.

[12] Aldana J., Wang Y.A., Peng X. (2001). Photochemical instability of CdSe nanocrystals coated by hydrophilic thiols. J. Am. Chem. Soc.

[13] Volkov Y., Mitchell S., Gaponik N., Rakovich Y.P., Donegan J.F., Kelleher D., Rogach A.L. (2004). *In-situ* observation of nanowire growth from luminescent CdTe nanocrystals in a phosphate buffer solution. Chemphyschem.

[14] Pellegrino T., Kudera S., Liedl T., Muñoz Javier A., Manna L., Parak W.J. (2005). On the development of colloidal nanoparticles towards multifunctional structures and their possible use for biological applications. Small.

